# Using participatory risk analysis to develop a song about malaria for young children in Limpopo Province, South Africa

**DOI:** 10.1186/s12936-018-2320-7

**Published:** 2018-04-27

**Authors:** Chad M. Anderson, Cheryl M. E. McCrindle, Taneshka Kruger, Fraser McNeill

**Affiliations:** 1 Faculty of Health Sciences, School of Health Systems and Public Health, University of Pretoria, Pretoria, South Africa; 2 Department of Anthropology and Archaeology, Faculty of Humanities, University of Pretoria, Pretoria, South Africa

**Keywords:** Malaria prevention, Preschool children, Risk communication, Tshivenda music, Songs, Participatory action research, Participatory risk analysis

## Abstract

**Background:**

In 2015, malaria infected over 212 million people and killed over 429,000 individuals, mostly children under 5 years of age, with 90% of malaria cases occurring in sub-Saharan Africa. The aim was to develop an age and culturally appropriate song for Tshivenda-speaking children under 5 years of age to decrease the risk of malaria in Limpopo Province, South Africa.

**Methods:**

Document review was used to identify appropriate disease determinants to decrease risk in children < 5 years old in the study area. These were used to develop lyrics and music for a song about malaria in line with the principles of participatory risk analysis. The age and cultural appropriateness of the song as well as disease determinants chosen were reviewed using a modified Delphi technique, by 10 purposively selected experts in malaria (4), Vhavenda music (3) and early childhood education (3). Thereafter, the song was translated into Tshivenda and reviewed by two focus groups living in the study area, one including female caregivers and pre-school teachers (n = 7) and a second comprising of male community based malaria control personnel (n = 5).

**Results:**

The experts surveyed and both focus groups strongly supported the inclusion of knowledge about the link between mosquitoes and malaria and that children should know the signs of malaria to facilitate early diagnosis. Although the expert group felt that bed nets should not be mentioned, both focus groups suggested the inclusion of bed nets and it was observed that community members were purchasing their own nets. Focus group members also felt that young children should not be involved in internal residual spraying initiatives.

**Conclusions:**

It was concluded that although risk communication on malaria prevention and treatment in young children should be aimed at caregivers, an age and culture appropriate song about malaria could be developed to help young children protect themselves. This song focused on understanding the link between mosquitoes and malaria, preventing exposure and recognising signs of disease.

**Electronic supplementary material:**

The online version of this article (10.1186/s12936-018-2320-7) contains supplementary material, which is available to authorized users.

## Background

**Fig. 1:**
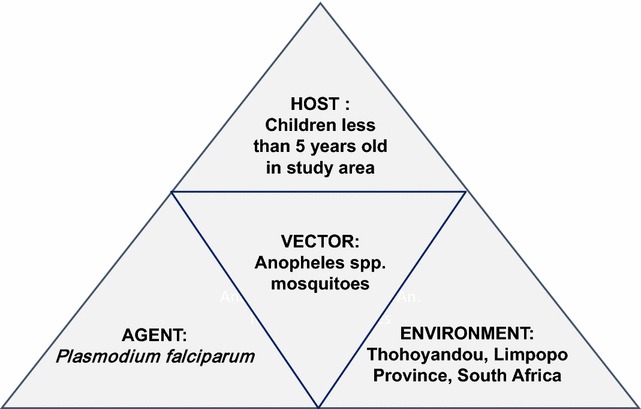
Epidemiological triad for malaria in young children in the study area (modified after Arshad et al. [4])

Malaria killed 429,000 people in 2015, with 90% of malaria cases occurring in sub-Saharan Africa. A large proportion of deaths from malaria occur in children less than 5 years of age as they have not yet acquired immunity [1]. In South Africa there is a risk of malaria in the north eastern parts of Limpopo, Mpumalanga Kwazulu-Natal Provinces [2]. Epidemiological factors are called disease determinants and the interaction between the agent, host and environment plays a role in the likelihood of disease occurring [3]. This interaction is often called the “epidemiological triad” and Fig. 1 also reflects this triad for malaria and interaction with a mosquito vector in the study area [4].

In South Africa, the agent of malaria is mainly the blood parasite *Plasmodium falciparum* and it is transmitted to the human host by mosquitoes belonging to the *Anopheles gambiae* and *Anopheles funestus* complexes, mainly the species *Anopheles arabiensis* and *An. funestus* [5, 6]. Human behaviour is also a disease determinant influencing the morbidity and mortality of malaria in the host [7]. Locally specific environmental determinants and climatic conditions, particularly high humidity and rainfall, are crucial to understanding and control of malaria in South Africa. Malaria is a highly seasonal disease regarded as endemic in the study area, Thohoyandou, in the north east of the Limpopo Province [6]. The location of the study area is reflected on the malaria risk map [8]. The Limpopo Provincial Malaria Control Programme (MCP) includes in its policies the use of indoor residual spraying (IRS) [9]. However early diagnosis and lack of knowledge in endemic areas are still the main constraints to eradication [5, 6, 9]. It was strongly motivated that by 2018, 100% of the population at risk for malaria in South Africa, should have adequate knowledge, attitudes and practices in place, through access to appropriate information, education and communication [9].

**Fig. 2:**
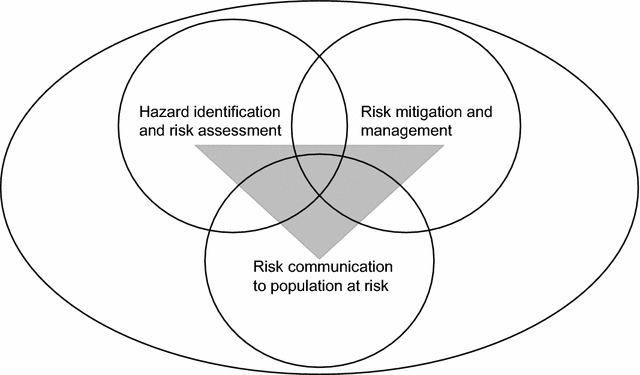
Participatory risk analysis framework (modified after Roesel et al. [17])

A risk based approach is considered appropriate for prevention and management of vector borne diseases [10]. The risk of contracting malaria in South Africa can be significantly reduced by preventing mosquito bites, even in low risk areas [2, 7]. Participatory risk analysis can be used to reduce the risk of disease using three phases: magnitude and frequency of disease exposure can be estimated using risk assessment, mitigation strategies can be developed and the risk reduced by appropriate risk communication [11, 12]. These three stages are illustrated in Fig. 2.

Early childhood programmes aim at instilling life-long learning in children using conceptual and cognitive learning strategies [13]. According to Piaget’s theory of early childhood development, during the pre-operational phase (2–7 years of age), young children begin to use language, memory and imagination [14]. In both European and African culture, this is the time when grandmothers use traditional nursery rhymes and songs to convey information to young children [15]. In South Africa, children are carried on their mother’s backs until about 2 years of age, then they are able to run about and it is at that stage that caregivers (like mothers, older sisters, aunts or pre-school teachers) start to teach them life skills. Young children like these, who are at risk of malaria, are pre-literate, but communication can be facilitated through the medium of songs and dance to enhance learning [13, 16]. Music and song are used in advertising to significantly increase recall and comprehension of a product with songs or “jingles” specifically targeted at the audience likely to purchase that product [17]. Musical interventions have previously been used to prevent or manage disease through promoting behaviour change in adolescents and adults, but no examples were found for young children [18, 19]. Examples in Africa include HIV/AIDS peer group education amongst young women, songs on preventing Ebola, as well as a song for stamping out malaria [20–22]. It appears, that there is a gap in published knowledge about children less than five being a target audience for risk communication strategies and this may be a new way to improve public health interventions aimed at reducing malaria. The aim of this study was to identify disease determinants appropriate to reducing the risk of malaria in young Tshivenda speaking children in Limpopo and use these as lyrics in an age and culture appropriate song.

## Methods

### Study area and target population

The study area was Thohoyandou, which is the administrative centre for the Vhembe District Municipality and Thulamela Local Municipality in Limpopo Province in South Africa [8]. The study population were malaria experts and caregivers for Tshivenda speaking children less than 5 years of age, in communities where traditional songs for children were commonly used. Risk communication messages about dates of IRS, malaria prophylaxis medications, topical repellants, provision of bed nets and protective clothing, or taking sick children to a clinic, must be addressed to the adult caregivers in the study area. Clinic personnel informed caregivers like parents, grandmothers or teachers (but not young children) about malaria. The children themselves were the population at risk, but could not be involved in the study for ethical reasons.

### Procedure

The study was based mainly on qualitative methods, applicable to participatory action research and risk analysis [11, 12]. Literature was reviewed using a document search to identify determinants of malaria in children less than 5 years of age, that could be used to reduce the risk of malaria in the study area. These determinants were used to develop the lyrics for an age and culturally appropriate song in the Tshivenda language. The music was conceptualized during a participatory workshop with four Venda musicians and a Venda cultural expert (who is also a recording musician) in the Department of Anthropology and Archaeology at the University of Pretoria, South Africa.

The initial and revised songs recorded, were evaluated using a plan-do review (action research) expert opinion survey, similar to the Delphi technique [23]. Ten participants were purposively selected for their expertise in malaria (four academics), music (four musicians) and preschool education (four academics). Expert opinions on the lyrics and music were scored using a five point Likert scale [24]. After the first round, changes were made and consensus was reached after a second round. Ten questions were asked initially, then the same questions put to the same experts in the second round.

The questions asked were:

1. Are the music and words of the song culturally appropriate for Thohoyandou?
2. Are the music and words of the song age appropriate for children < 5 years of age in Thohoyandou?
3. Is encouraging children to sleep under mosquito nets, appropriate to help reduce the risk of malaria in children > 5 years old in Thohoyandou?
4. Is knowledge about IRS appropriate to help reduce the risk of malaria in children < 5 years old in Thohoyandou?
5. Is encouraging children to wear long sleeves and socks at night appropriate to help reduce the risk of malaria in children < 5 years old in Thohoyandou?
6. Is encouraging children to recognize and reduce stagnant water sources appropriate to help reduce the risk of malaria in children < 5 years old in Thohoyandou?
7. Is informing children that mosquitoes are the vector for malaria an appropriate way to help reduce the risk of malaria in children < 5 years old in Thohoyandou?
8. Are these five disease determinants enough to help reduce the risk of malaria in children < 5 years of age in Thohoyandou, or should more be added?
9. Are the words used in the song clear and is it easy to understand what is being said?
10. Is song easy to remember and easily repeatable for children < 5 years of age to teach others the song?

* Comments and suggestions ________________________________

The lyrics were initially written in English for evaluation by the experts, then translated into Tshivenda for evaluation and discussion with two focus groups in the study area.

Focus groups are considered very useful for obtaining subjective opinions from key stakeholders [25]. The first focus group (n = 7) were all female caregivers and included Tshivenda speaking village mothers, grandmothers and pre-school teachers in the study area. The second focus group (n = 5), was comprised of malaria management and spray-control personnel, working for the Limpopo Province malaria control programme (MCP). These were all Tshivenda speaking men, with knowledge about malaria prevention at community level in the study area. Music and lyrics were changed in line with feedback from both focus groups as well as the expert opinion surveys.

The Likert-scale data obtained from the expert opinion surveys was analysed using observational (categorical) statistics. In contrast, the analysis of focus groups was a narrative review of recordings made with permission from respondents. Triangulation of information obtained from analysis of data from the document search, expert opinion surveys and focus group discussions was essentially a qualitative approach to data validation.

## Results

### Document review of disease determinants

Table 1 shows a list of disease determinants obtained through document review. The numbers in the second column refer to the citation list at the end of this paper.

**Table 1:** Disease determinants relevant for reducing exposure and risk of malaria, that were identified for inclusion in song lyrics

Determinant	Citation number^a^
Knowledge that mosquito bites cause malaria	[2, 7]
Internal residual spraying in village homes	[2, 6, 9]
Preventing mosquito bites at night	[5, 27]
Pools of stagnant water	[22, 29, 31]
Using bed nets at night	[5, 26, 29]
Early recognition of the signs of malaria	[5, 6, 27]
Mosquito repellents	[5, 27, 28]
Protective clothing (feeding sites of vector)	[27, 30]

^a^The number quoted is the number of the citation in the citation list

These disease determinants were included in the lyrics of the first draft of the proposed song, as they could be influenced by changing the behaviour of the population at risk (young Tshivenda speaking children in the study area).

The lyrics of the first song were:

- *SLAP*
- *Slap, slap, slap the mosquito*
- *SLAP! SLAP! SLAP!*
- *Dirty water? Throw it away!*
- *Don’t let mosquitoes, breed today!*
- *Wear your pants and long sleeves,*
- *Don’t let mosquitoes, bite your knees!*
- *Slap, slap, slap the mosquito! SLAP! SLAP! SLAP!*
- *When it’s dark, stay inside,*
- *HIDE from mosquitoes, hide, hide, hide!*
- *Close the window, close the door,*
- *Keep the mosquitoes, out for sure!*
- *Slap, slap, slap the mosquito! SLAP! SLAP! SLAP!*
- *Sleep in the net, on your bed,*
- *Don’t let mosquitoes bite your head!*
- *Spray those mosquitoes, SPRAY! SPRAY! SPRAY!*
- *Spray those mosquitoes ‘til they’re DEAD!*
- *Shhhh, shhhh,*
- *NO MOSQUITOES? YAY!!!!!!*

### Delphi technique for expert opinion survey

During the first round of expert opinion surveys, 8 of the 10 experts believed the song needed to be reviewed, as it was sung too fast and the words were not clear. Two early childhood experts were happy with the song. A malaria expert pointed out that *Anopheles* mosquitoes fly silently, so the “*shhh, shhhh*” listening for mosquitoes was not appropriate. One of the education experts stressed that for younger children, repetition was needed and using short understandable sentences would help. All four of the malaria experts said that nets should not be included in the lyrics. As free nets provided by the state were not being used, they felt internal residual spraying was more important. All experts agreed that children learning that mosquitoes caused malaria was very important, as this knowledge was lacking in rural communities. They mentioned that this was not sufficiently emphasized in the first song. The malaria experts also suggested including early signs of malaria and that children should tell parents or teachers if they felt sick. Fans and smoke to repel mosquitoes were also mentioned by one musician, who lived in the study area.

After modifications and editing, the song was re-recorded in English and played to the experts during the second round of interviews. Bed nets were excluded from the lyrics, whereas using smoke and fans was included. Reporting signs of illness to an adult was also included. During the second round of interviews, one malaria expert disagreed with the message about socks and long sleeves, as he said children in rural areas did not own these sort of clothes and it was too hot to wear them. However, the experts reached consensus, approving the amended lyrics and music, but suggested that the song was too long for children less than 5 years of age and suggested translation into the vernacular. One of the musical experts really enjoyed the music, saying:

*“… it’s so catchy it will stay in my head…”*

**Fig. 3:**
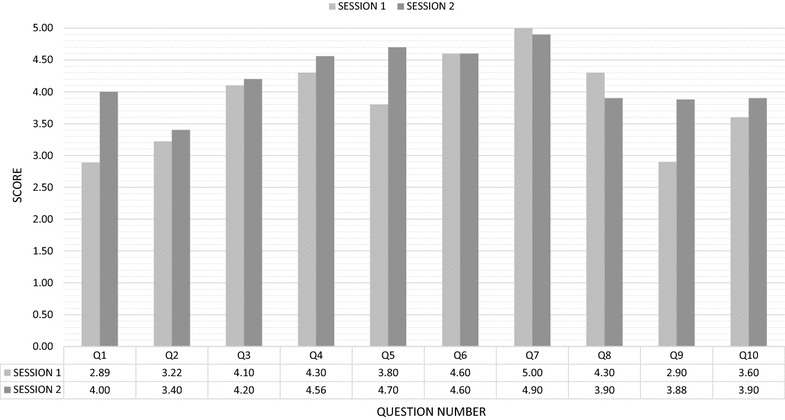
Likert score means comparing the two rounds of interviews with the experts

A comparison of the mean Likert scores for the 10 questions asked of the 10 experts in both rounds, is shown in Fig. 3.

### Focus group discussions in the study area

Following the second round of expert interviews, the song was translated into Tshivenda. The song was then workshopped by the musicians and they adjusted the tempo as well as simplifying the lyrics and making changes to disease determinants. They quickly established that a certain style of drumming would need to be selected, as traditional Vhavenda music had a specific rhythmic component and the speed needed to be adjusted so small children could dance or move to the beat. It was also agreed that repetition should be central to the song structure. Every verse, would start with ‘*nne ndi vhunyunyu*’ (I am a mosquito), and end with ‘*ndi na malaria*!’ (I have malaria!).

The full-length song (for children older than 5 years of age) as well as a simplified version that included fewer verses (for children younger than 5 years of age) were recorded. The songs were then played to the two focus groups in the study area. The overall feedback from both focus groups was unanimously positive. Many of the members of the first group (mothers, teachers and grandmothers) started joining in singing and chanting to the beat of the song. One mother had a young child about 3 years old with her and her little boy started dancing to the song, with the encouragement of the mothers and grandmothers!

One mother said, “*We can’t wait to hear this on the radio*.”

Another said, “*I think it’s so import to start teaching kids early about these things through music; maybe more things could be done like this in the future.*”

Neither group, however, was positive about indoor residual spraying (IRS) being included in the song. Group one (caregivers) said that if they were not home they did not want children letting strangers into the house. Group two, some of whom were officials responsible for IRS in the study area, were also worried about young children coming into contact with the poisons being sprayed, they said that generally they sent children far away during spraying.

Both of the focus groups believed that adding the symptoms of malaria was important to early diagnosis. This would help children to tell their parents or caretakers they may have malaria if they knew what it felt like. All respondents in both focus groups felt the song and music were both culture and age appropriate. One preschool teacher in the first group said: *“Well Yes! The repetition is important I think. This is going to make children learn faster, and I like how it connects mosquitoes to malaria.”*

One of the musicians involved in developing the song, helped facilitate the focus group meetings, as he came from the study area and spoke fluent Tshivenda. Few members of the first focus group spoke English fluently, so he was able to explain the questions and sing parts of the song in the vernacular, as well as modifying the lyrics in accordance with the suggestions of focus group members. Respondents talked to him freely in Tshivenda about the changes they wanted and modified words and phrases so they would be easily understood by village children. The members of the first focus group felt strongly that sleeping under mosquito nets should be added to the song. This came as a surprise, as the experts had said otherwise.

Caregiver 4 (a grandmother) said, *“I think adding nets to the song is very important”*

Researcher replied, *“Oh! Do you have nets in your house currently?”*

Caregiver 4, answered, *“EH! I have!*

All respondents were then asked who used nets at home. Four of the seven women currently used nets currently in their houses. One noted that she understood that she needed a net, she just had not purchased one yet. After the first focus group discussion, caregiver 4 led the way to her house where she showed everyone her mosquito nets. They were not obtained free from the government, the village women purchased them from a small village shop nearby, called “The China Shop”.

During informal discussions with the first focus group members, all seven agreed strongly that malaria symptoms should be added to the song so that sick children could be taken quickly to the clinic for treatment.

Caregiver 1 said*, “I think symptoms are important for children to understand, so they can tell their parents when they are feeling sickness.”*

Following this, it was decided to add verses about bed nets and symptoms to the song lyrics. The musician asked the advice of the village women in the first focus group, on how best to include these ideas in the lyrics. The women suggested Tshivenda words that small children would understand easily and that made sense. They also sang them, to show the musician how to incorporate them in the rhythm and beat of the traditional music. This was truly a participatory research approach, as women in the study area are adept at improvising songs about village life and the young musician grew up in the same area.

The first focus group agreed unanimously with the words of the song, after he had changed them. All participants agreed that the words were no longer ‘complex’ or ‘deep’ Tshivenda terms; and they liked the repetition of the first and last lyric. It was felt that even if the children might not learn all of the lyrics initially, they would understand and sing along with those being repeated throughout the song. When asked if the song was age and culture appropriate, two members of the first focus group commented:

Caregiver 1 (a mother) said, “*Yes! It is! And I think that all children will be able to learn it fast*.”

Pre-school teacher 1 said, *“Well Yes. The repetition is important I think.” “This is going to make children learn faster like she said, and I like how it connects mosquitoes to malaria.”*

During the song improvisation, those from the first focus group also suggested adding motions and actions to the lyrics. They thought children would learn better, if actions were attached to some of the words. Caregiver 4, who was a pre-school teacher, even suggested:

*“Umm…what if this were paired with a game?”*

The possibility of a game about malaria, attached to the song, had also been brought up by one of the experts from the early childhood education sector consulted during the opinion surveys.

The second focus group, which included only adult male respondents from the MCP, did not contribute as much as the first group. However, the questions and answers, that were relevant to the song process, are narrated below.

The researcher asked, *“Are the determinants used in the song the most important, or would you use different determinants to prevent malaria for children under the age of 5?*

Second group, member 2, answered *“The only thing I see missing would be the symptoms. I think that it would be ok to add them so that people can understand, even if it’s just fever or headache so they can understand. Sometimes flu can be misunderstood.*

Second group, member 3, contributed, *“I think that IRS is too much for children. You know some people have stigma against the sprayers and they don’t let them spray. So I think maybe the song should just talk about accepting the sprayers but not discuss spray because maybe the children will get the doom spray thinking its IRS and then that child could harm himself, just because he wants to help out.”*

The use of bed nets for young children was also suggested by the second focus group. All members of the second focus group agreed the song was culturally appropriate and they said they liked it. As the first focus group had also felt negative about IRS, suggested the use of bed nets and wanted the children to recognize the signs of malaria, these three suggestions, were subsequently incorporated in the lyrics.

On returning from Thohoyandou, the group of musicians and the Venda cultural expert, convened in a recording studio in downtown Pretoria and reworked the changes to the lyrical content. The *Zwidade* rhythm was programmed into a computer, using recording software, and an appropriate tempo was set accordingly. The repetitive structure at the start and end of each verse was kept, but it was decided that for maximum impact and potential participation for children who might not remember all the lyrics, the final line of each verse (*ndi na malaria*!—I have malaria!) should include a multitude of voices, giving the aural impression of a sing-along, with loud hand-clapping. The final song is included as a sound byte (Additional file 1). In the Tshivenda language, a singular mosquito is *lunyunyu*. In the plural, this becomes *vhunyunyu*

The final lyrics of the malaria song were:

(The English version was translated verbatim from Tshivenda)

**Verse 1**

*Nne ndi lunyunyu*, I am a mosquito
*Nne ndi a luma*, I bite
*Athi funi vhathu*, I don’t like people
*Ndi na Malaria!*: I have malaria!

**Verse 2**

*Nne ndi lunyunyu,* I am a mosquito
*Ndi da na malwadze,* I come with diseases
*Thoho iya rema,* I gave you a headache
*Ndi na Malaria*!: I have malaria!

**Verse 3**

*Nne ndi lunyunyu,* I am a mosquito
*Ndi da na malwadze*: I come with diseases
*Dzungu na mufhiso*: Dizziness and fever
*Ndi na Malaria!*: I have malaria!

**Verse 4**

*Nne ndi lunyunyu,* I am a mosquito
*Madini o imaho,* I live in still water
*A tevhuleni kule*: throw it far away
*Ndi na Malaria!*: I have malaria!

**Verse 5**

*Nne ndi lunyunyu*, I am a mosquito
*Duvha li tshi kovhela*: at sunset
*Fukani muvhili*: cover your body
*Ndi na Malaria!*: I have malaria!

**Verse 6**

*Nne ndi lunyunyu*, I am a mosquito
*Shumisani nethe,* use a net
*Nne ndi si dzhene*: so that I can’t get in
*Ndi na Malaria!*: I have malaria!

**Verse 7 (×4)**

*Ri vhana vha Afrika*: We are the children of Africa
*Ro guda luimbo*: we learned this song
*Ro pandela vhunyunyu*: we chased away the mosquitos
*Ha Malaria!*: Mosquitos with malaria!

All the above were then repeated.

## Discussion

Although several publications agree that communication of knowledge about malaria is very important in preventing malaria in endemic areas, specifics on what sort of information on malaria should be conveyed to populations at risk, are not consistent [5–7, 9, 26–29]. In this study, however, both the experts and focus group members agreed strongly that the link between mosquitoes and disease very important knowledge for young children living in malaria endemic areas. Also emphasized by both the expert opinion survey and both focus groups, was that children should know the signs of malaria. This underscores that both experts and community caregivers, recognized the value of early diagnosis and treatment in saving children’s lives. It was also very interesting that community members interviewed during focus group discussions in the study area, differed from experts on the importance of bed nets. Another difference of opinion was the importance of IRS, where members of both focus groups felt that young children should not be encouraged to let strangers into their homes or be present during spraying.

It was mentioned in the background to the study and in Table 1 high rainfall and humidity are environmental disease determinants for malaria. They promote multiplication of the vector, especially in pools of stagnant water [30, 31]. Key informants suggested that bed nets were important for malaria control, although experts felt that indoor IRS was more important. The caregivers showed that they did not have be dependent only on state sponsored spraying, but also spent their own money on bed nets. The importance of bed nets as a part of malaria prevention, was emphasized after the study was completed, when unforeseen heavy rainfall in late summer, during the 2016/17 season, resulted in budget constraints for indoor IRS. The vector population increased rapidly and unexpectedly, resulting in an epidemic [32]. It appears therefore, that the participatory risk analysis method proposed in this study for developing a song for preschool children, might also be useful to develop other risk communication strategies and align them to risk priorities in different target populations under changing conditions.

**Fig. 4:**
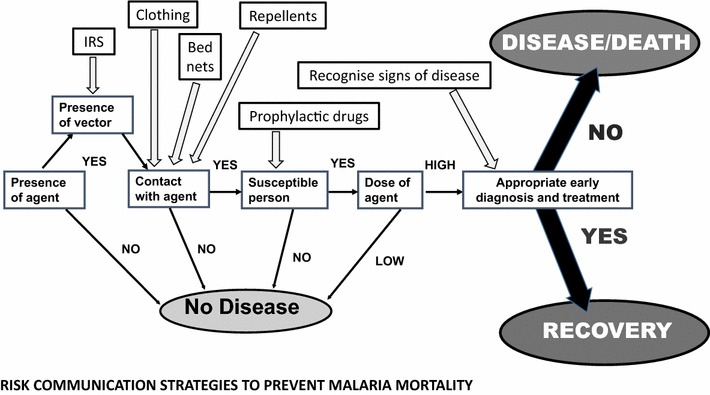
Appropriate risk communication opportunities along the risk pathway for malaria

Using a risk based approach, it is proposed that the following risk pathway (Fig. 4) could assist in deciding the opportunities for risk communication that are appropriate along the risk pathway for malaria.

The population at risk in the current study was children under the age of 5 years. In the study area, risk communication to this population was mediated through adult caregivers. However, this study proposes that young children can also be empowered to protect themselves, using knowledge gained through a song. Similar to life skills gained through nursery rhymes, the song encourages early learning about the dangers of being bitten by mosquitoes, the use of bed nets and fans and the signs of malaria. It is also recommended, in line with UNICEF suggestions for communicating with young children [33], that the song be accompanied by movements and dancing; or a game where one child dressed as a mosquito chases others, who, if caught, lie down and pretend to be sick. Both of these additions were suggested by preschool teachers during the focus group discussions with care-givers. Early childhood education is based on the theory that life skills learned by children under 7 years of age remain and are passed on in turn to their own children when they are adults. This song could be a partial solution to the lack of knowledge about malaria in rural communities highlighted in several publications. It is recommended that participatory risk analysis methods described in this study, could also be used to develop songs with specific messages about malaria prevention, appropriate to language, culture and age of young children in other areas where malaria is endemic.

## Limitations

This study was conducted to explore issues around malaria prevention practices in which children less than 5 years of age could participate. For ethical reasons, children could not be asked any questions. Consequently, opinions were sought from experts in the field of study as well as members of the community. Malaria vulnerability can be due to biological, cultural, socioeconomic, and environmental factors and can influence community participation in malaria prevention practices. This song developed through a participatory risk analysis framework, investigated the possibility of communicating the risk of malaria to a susceptible population, by taking into account the host characteristics, including behaviour. However, it is beyond the scope of this research to explore the efficacy of this particular intervention. Further investigation into the impact on the community participation for malaria elimination could occur at sentinel sites for community participation.

These results are limited in generalizing for the population of the study area in South Africa. The authors recognize that the focus groups may have had social desirability bias, as some members want to show they knew about malaria prevention programmes. The study was done in a malaria research area where malaria education initiatives exist. Finally, there might have been be some loss of information during translation of Tshivenda to English, sound loss on the recorders, or misunderstanding from the focus group note recorder.

## Conclusions

It is concluded that a culturally and age appropriate song to help children under the age of 5 years old has been created and accepted by selected representatives of the Tshivenda-speaking community and malaria experts in the study area. All of the determinants were agreed upon and that the use of these determinants could bring behavioural changes to the young children. The complete song is now ready to be made into a video. Further studies to measure the effectiveness of the song are recommended.

## Additional file

**Additional file 1.** The final recorded song about preventing malaria, for young children in Tshivenda speaking communities in Limpopo Province, South Africa. (Sound-byte).

## Abbreviations

WHO: World Health Organization
IRS: Indoor residual spray
CDC: Center for Disease Control
MCP: Malaria Control Programme
